# NEXGB: A Network Embedding Framework for Anticancer Drug Combination Prediction

**DOI:** 10.3390/ijms23179838

**Published:** 2022-08-30

**Authors:** Fanjie Meng, Feng Li, Jin-Xing Liu, Junliang Shang, Xikui Liu, Yan Li

**Affiliations:** 1 School of Computer Science, Qufu Normal University, Rizhao 276826, China; 2 Department of Electrical Engineering and Information Technology, Shandong University of Science and Technology, Jinan 250031, China

**Keywords:** drug synergy prediction, network embedding, cancer, drug combination

## Abstract

Compared to single-drug therapy, drug combinations have shown great potential in cancer treatment. Most of the current methods employ genomic data and chemical information to construct drug–cancer cell line features, but there is still a need to explore methods to combine topological information in the protein interaction network (PPI). Therefore, we propose a network-embedding-based prediction model, NEXGB, which integrates the corresponding protein modules of drug–cancer cell lines with PPI network information. NEXGB extracts the topological features of each protein node in a PPI network by struc2vec. Then, we combine the topological features with the target protein information of drug–cancer cell lines, to generate drug features and cancer cell line features, and utilize extreme gradient boosting (XGBoost) to predict the synergistic relationship between drug combinations and cancer cell lines. We apply our model on two recently developed datasets, the Oncology-Screen dataset (Oncology-Screen) and the large drug combination dataset (DrugCombDB). The experimental results show that NEXGB outperforms five current methods, and it effectively improves the predictive power in discovering relationships between drug combinations and cancer cell lines. This further demonstrates that the network information is valid for detecting combination therapies for cancer and other complex diseases.

## 1. Introduction

In cancer therapy, it is difficult to provide effective treatment using a single drug [1]. However, drug combinations can increase therapeutic efficacy and reduce the toxic effects of drugs by targeting multiple molecular mechanisms in cancer cells [2,3]. At the same time, drug combination therapy also shows great potential in overcoming drug resistance [4]. For cancer patients, choosing the right combination of drugs to improve the efficiency of their treatment can greatly reduce their suffering. Traditional drug combination identification methods often consider clinical trials or high-throughput screening (HTS) [5], but they still have many problems. The time and money spent on clinical trials are unknown, and these methods are prone to errors and may even expose patients to harmful treatments, so it is not an effective way to identify large numbers of drug combinations [6]. Recently, HTS has been widely used to identify effective combinations in preclinical settings [7]. The method measured a large number of drug combinations at different doses and was applied to different cancer cell lines [8]. Compared with clinical trials, HTS can identify drug combination in a reasonable time through multiple drug combination databases [6,9]. However, with the dramatic increase in data volume, it is impractical to use HTS to consider all combinations [10,11]. Therefore, more effective methods are needed to explore the drug combination space, improve efficiency, and reduce errors as much as possible.

With the progress of computer technology, the machine learning model has been developed to explore drug combinations for cancer diseases [12]. More and more biological knowledge has been used in the prediction models of machine learning. Peng et al. integrated the molecular and pharmacological features of drugs using a Bayesian network [13]. Cheng and Zhao applied five algorithms (naive Bayes, decision tree, k-nearest neighbor, logistic regression, and support vector machine) and employed four features based on drug–drug similarity to identify the drug combinations [14]. Yu et al. applied similarity networks to predict drug–target interactions and demonstrated the feasibility of predicting drug combinations through network embeddings [15,16]. Advances in biological technology have provided abundant biological data, with a huge amount of data available. Recently, in order to utilize biological information, deep learning has begun to be applied to drug synergy prediction. Kristina and colleagues used chemical and cancer genomic information to construct a new model (DeepSynergy) to predict combinations of anticancer drugs for multiple types of cancer [17]. MatchMaker, proposed by Halil et al., combines the chemical features of each drug with the gene expression feature of the cell line [18]. TranSynergy, proposed by Liu et al., uses PPI network information and gene profiles to construct the features of drugs and cell lines. Notably, they applied the random walk with restart algorithm (RWR) in the network to infer drug representations [19]. However, they considered drug target genes but ignored the topology between drug targets and disease proteins in PPI networks, which also have complex biological interactions. In recent network science research, the relationships between and importance of drug targets and disease proteins have been investigated through cluster detection algorithms [20] and drug–disease proximity measures [21]. Cheng and his colleagues quantified the relationship between drug targets and disease proteins in the human protein–protein interaction group, and they found that all possible drug–drug–disease combinations can be divided into six topologically different categories [22]. This confirmed the predictability of considering potential drug–drug–disease relationships through a PPI network. Graph convolutional neural networks and self-attention mechanisms have been applied in the fields of predicting frequently occurring diseases and predicting compound–protein interactions [23,24]. Yang et al. proposed a method based on a graph convolution network (GraphSynergy), which utilized proteins related to drug targeting and specific cancer cell lines [25]. However, the application of existing network science methods to drug combination therapy still has a large space for exploration.

In this work, we propose a novel framework, extreme gradient boosting for network embedding (NEXGB), that utilizes PPI network information to identify drug combinations with synergistic effects in specific cancer cell lines. NEXGB combines the struc2vec [26] component, which can effectively capture the topological information of the target protein to generate drug features and cancer cell line features [21,22]. Two targets with the same local network structure should have the same potential features. Therefore, we obtain the drug features and cell line features from the potential information of targets and protein information. Then, the obtained features are put into XGBoost to identify drug combinations with synergistic effects in cancer treatment [27]. To validate the effectiveness of NEXGB, we apply NEXGB on two datasets: a recently developed unbiased oncology compound screen (Oncology-Screen) [28] and a large drug combination dataset (DrugCombDB) [29]. We also compare NEXGB with other recent advanced methods to further illustrate the performance of NEXGB.

## 2. Results

In this part, we list the key parameters of the model, illustrating the effect of the feature dimension on model performance. Furthermore, we demonstrate the superiority of the NEXGB model and its predictive performance in specific tissues and specific cell lines.

### 2.1. Parameter Relation

In this study, struc2vec is used to generate feature vectors for proteins. We set the length of random walk sequences to 80, and each node generates 20 random walk sequences. Finally, a 64-dimensional vector is generated for each gene through the Skip-Gram model. Table 1 provides the relevant parameters of this part. The XGBoost model performs subsequent classification training. The key parameters are listed in Table 2. We apply the grid search method to adjust the parameters of this part to find the optimal parameters possible.

### 2.2. Comparison Study of Existing Methods

We evaluate performance on two recently developed large drug combination datasets, Oncology-Screen [28] and DrugCombDB [29], and use five metrics: accuracy (ACC), recall, area under receiver operating characteristic curve (AUC-ROC), area under precision-recall curve (AUC-PR), and F1 score. In this study, ACC represents the number of correct drug combinations predicted. Recall represents the probability of being predicted to be synergistic among all synergistic drug combination data. AUC-ROC is the area under the receiver operating characteristic curve calculated from the predicted scores. AUC-PR is the mean precision calculated from the prediction score. The F1 value combines the precision and recall score. The Oncology-Screen data provided synergistic information for 4176 drug combinations, covering 21 drugs and 29 tumor-associated cell lines. DrugCombDB is much larger and is the largest drug combination dataset by far, containing 764 unique drugs and 69,436 drug combinations in 76 unique cell lines. We compare the performance of NEXGB with other baselines. The compared methods include network proximity (NP [22]), matrix factorization (GraRep [30]), random walk (DeepWalk [31]), deep neural network (DeepSynergy [17]), and graph convolutional network (GraphSynergy [25]). NP quantifies the proximity between drug target proteins and disease target proteins by z-score and separation measures in the PPI network. The embedding dimension of GraRep and DeepWalk is 64. DeepSynergy utilizes related proteins as drugs and cell line features. The embedding dimension of GraphSynergy is 32 for Oncology-Screen and 64 for DrugCombDB.

We perform five-fold cross-validation on each of the two datasets. Cross-validation randomly splits the dataset and reports the average performance after repeating the experiment five times (Table 3). Our model outperforms all baselines on the Oncology-Screen dataset and shows decent performance on DrugCombDB. Specifically, both GraphSynergy and DeepSynergy are deep learning models specially designed for drug combination prediction tasks. The performance of DeepSynergy is mediocre, suggesting that the inability to capture graphical information may be the reason for this. The random-walk-based model (DeepWalk) outperforms the matrix-factorization-based model (GraRep) and the network-proximity-based model (NP), which indicates that capturing sufficient structural information can greatly improve the predictive ability of the model.

### 2.3. Discussion on Feature Dimension

**Figure 1:**
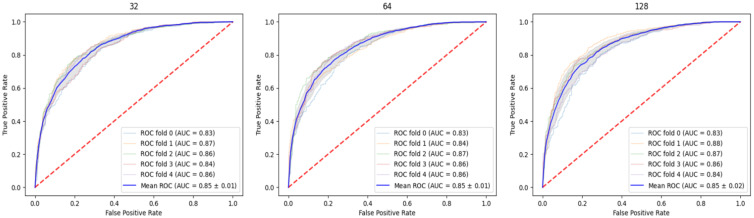
This figure shows the ROC curve and AUC value of the 32-dimensional, 64-dimensional, and 128-dimensional features of the node under the five-fold cross-validation. Among them, the blue curve is the average ROC and the corresponding mean, and the mean is around 0.85. Other colors correspond to the ROC curve and AUC value of each fold.

The output of our choice is a 64-dimensional vector of nodes. Therefore, the feature vectors for drugs and cancer cell lines are 64-dimensional, and the input to training is 192-dimensional drug combination–cancer cell line data. The struc2vec component can choose the dimension of the output node feature, and we output the 32-dimensional, 64-dimensional, and 128-dimensional node feature vectors through the PPI network. To illustrate the effect of feature dimension on the model, we train three different dimensions of features on the Oncology-Screen dataset. Through the ROC curve and AUC value, we find that the dimension of features has no significant effect on the performance of NEXGB. This proves that struc2vec has learned the features of the nodes, but the increase in dimension does not mean that the learned feature vector is correct and effective. Thus, in order to facilitate subsequent operations, we use all 64-dimensional feature vectors for experiments and explanations. The area under receiver operating characteristic curve (AUC-ROC) under five-fold cross-validation is shown in Figure 1. We run it five times in each of the three dimensions, and the average AUC values are all around 0.85.

### 2.4. Classification Method

**Figure 2:**
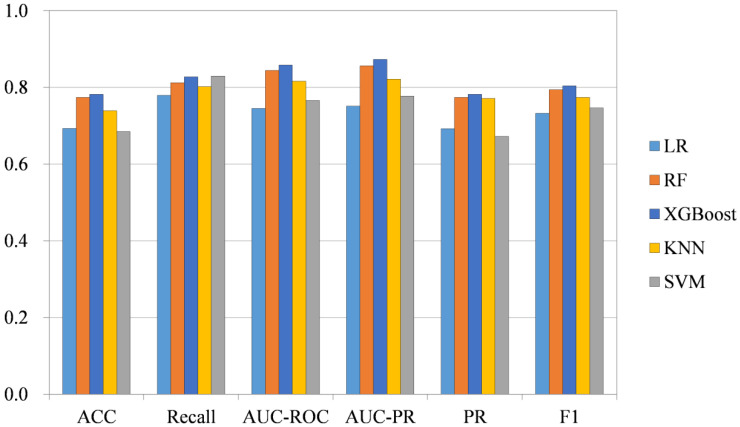
Classification method comparison. The five colors in the figure represent the five respective methods, the horizontal axis represents the performance index, and the vertical axis represents the corresponding value. Of the six indicators, XGBoost shows its superiority.

We apply the XGBoost method, which has been widely used in recent years, for classification and compare it with some classic machine learning methods: random forests (RF) [32], k-nearest neighbor (KNN), support vector machines (SVM) [33], and logistic regression (LR). In order to compare the differences between them and exclude the influence of other factors, the above methods use 64-dimensional feature vectors, and the parameters are tuned to train on the Oncology-Screen dataset. We use 6 performance metrics: accuracy (ACC), recall, area under receiver operating characteristic curve (AUC-ROC), area under precision-recall curve (AUC-PR), precision (PR), and F1 score. Each experiment was carried out 10 times, and the last 6 indexes were taken as the average value. We find that on the Oncology-Screen dataset, the recall value of XGBoost is not as good as that of SVM, but the other metrics are the best. Among the recall values, XGBoost is 0.827 and SVM is 0.829. Although the recall value of SVM is high, its performance on the other five metrics is not satisfactory. The results under the five-fold cross-validation are shown in Figure 2.

### 2.5. Prediction Performance on Specific Tissues and Specific Cell Lines

**Figure 3:**
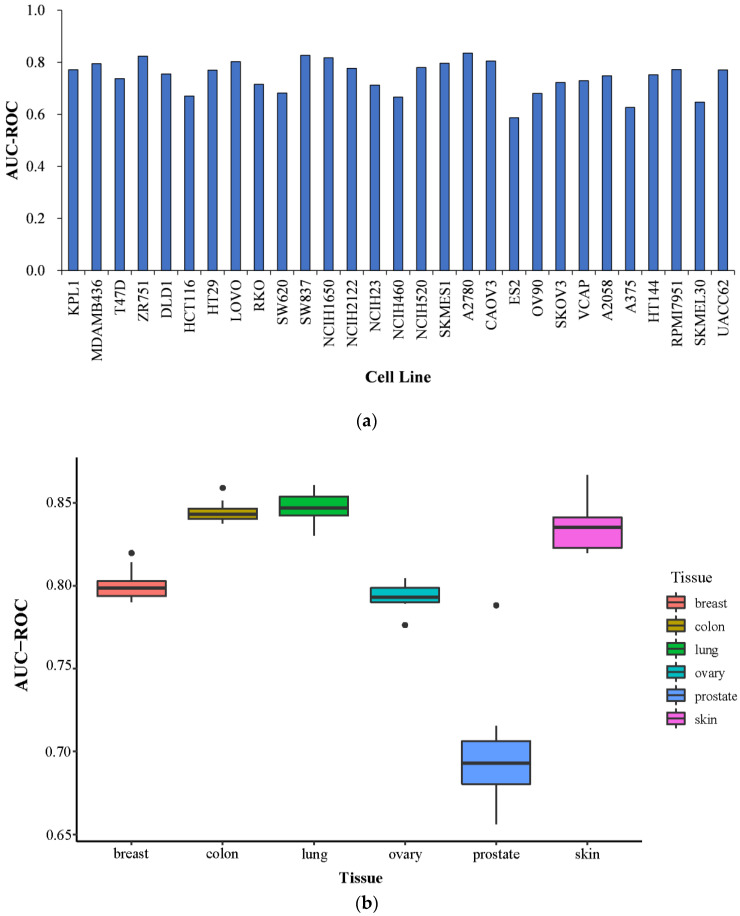
Cell-line-specific and tissue-specific prediction performances of NEXGB. (**a**) Cell-line-specific performance. The average ROC-AUC value in the 29 cell lines is around 0.75, with the highest value of 0.835 for A2780 cell line and the lowest value of 0.587 for ES2 cell line. (**b**) Tissue-specific performance. Among the six types of tissues, the prostate tissue had the lowest performance in general.

The predictive performance of the model is closely related to the associated proteins of the drug and cell line. We further investigate the performance of the NEXGB model on the Oncology-Screen dataset for specific cell lines and specific tissues. The performance is represented by the ROC-AUC value under cross-validation. Figure 3 shows the expression of NEXGB in different cell lines and tissues.

NEXGB has strong predictive power (ROC-AUC greater than 0.7) for over 75% of cell lines (Figure 3a). The ROC-AUC values of different cell lines range from 0.587 to 0.835. Among all 29 cell lines, the ES2 cell line, belonging to ovary tissue, has the lowest ROC-AUC value. The ES2 cell line has the fewest associated target proteins (56 associated target proteins), which may be one of the reasons for the poor performance of ES2 cell lines. However, the number of related target proteins is also less than 100, and DLD1 (75 related target proteins) belonging to colon tissue had a good performance. We believe that ES2 cell lines may be somewhat different from other cell lines (see Figure 3b). We also explore the performance of the NEXGB model on six different tissues: breast, colon, lung, ovary, prostate, and skin. The mean ROC-AUC values of the six tissues are 0.801 for breast cancer, 0.845 for colon cancer, 0.848 for lung cancer, 0.794 for ovarian cancer, 0.699 for prostate cancer, and 0.835 for skin cancer. Except for the prostate, the performance of NEXGB in the other five tissues is consistent. Among all tissues included in our data, prostate tissue has the lowest ROC-AUC value of all tissues. VCAP is the only prostate cancer cell line and underperforms among all cell lines. The target proteins associated with the VCAP cell line are the most numerous (830 target proteins) of all cell lines. The reason for the poor performance of prostate tissue may be related to the fact that it has only one cell line, VCAP, or that there are significant differences between VCAP and other cell lines. Table 4 shows the number of relevant target proteins of each cancer cell line.

**Figure 4:**
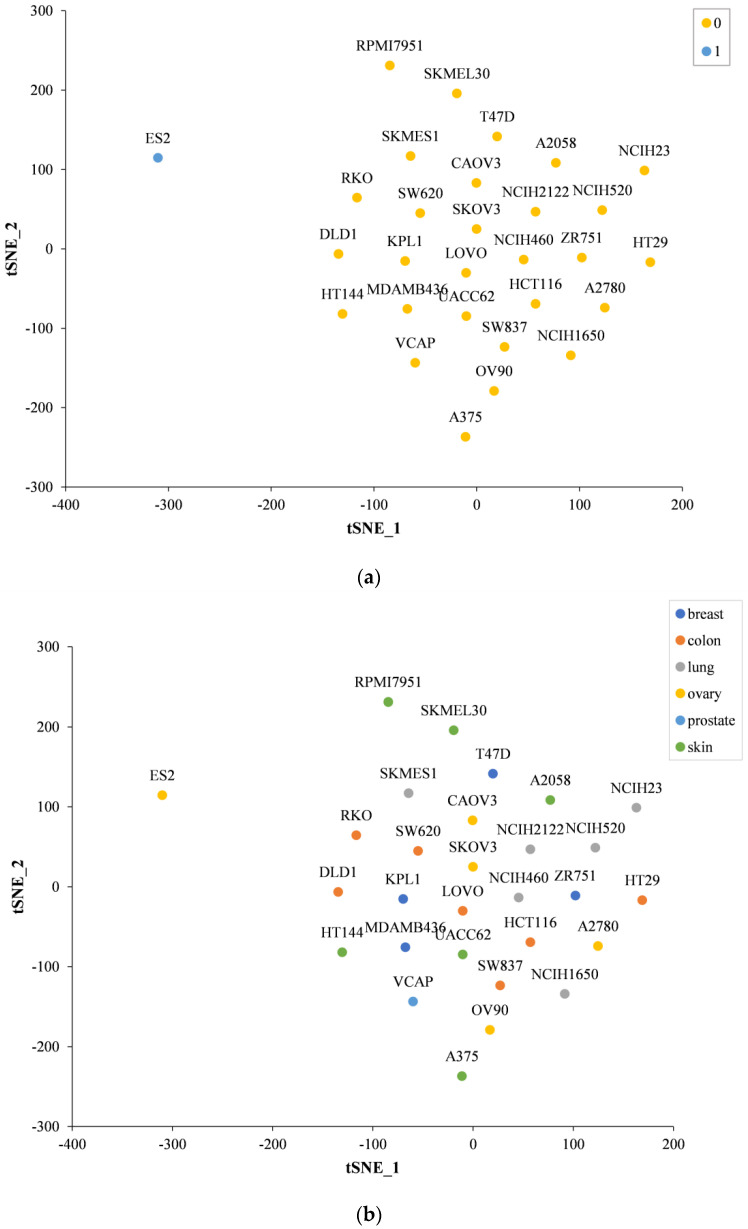
Different cell lines are subjected to t-SNE analysis to visualize the results. High-dimensional cell line vector representations are projected into 2D space with the first two t-SNE components. (**a**) Different colors indicate clusters per cell line assigned by the density-based spatial clustering of applications with noise (DBSCAN) clustering algorithm. (**b**) Different colors indicate different tissues of each cell line.

We performed t-SNE analysis on high-dimensional vector representations of cell lines, reflecting the relationships between cell lines in 2D space. Figure 4 shows that all cell lines are divided into two clusters by the density-based spatial clustering of applications with noise (DBSCAN) clustering algorithm. We find that the ES2 cell line belongs to a separate category, and the rest of the cell lines belong to another category. In addition, the VCAP cell line is on the fringes of a cluster. This suggests that both the ES2 cell line and the VCAP cell line may have unique characteristics.

Ovarian cancer is mainly classified into five histological types due to the characteristics of ovarian tissue: high-grade serous, low-grade serous, clear cell, endometrioid, and mucinous [34]. Ovarian clear cell carcinoma (OCCC) is a common ovarian epithelial cancer that accounts for 5% to 25% of ovarian cancers and has unique clinical and molecular features [35]. This may be one of the reasons why ES2 (which belongs to clear cell carcinoma alone) is different from other ovarian cancer cell lines in our dataset. In recent years, studies have shown that ES2 is different from other ovarian CCC cell lines. When the expression of hepatocyte nuclear factor 1β (HNF-1β) in ovarian CCC cell lines was examined using immunocytochemistry, intense nuclear staining was observed in OVMANA, OVISE, and OVSAYO, while no nuclear expression of NHF-1β was found in ES2 [36]. The unique characteristics of ES2 cell lines in ovarian clear cell carcinoma may be another reason for the unusual performance of ES2 cell lines in our dataset.

## 3. Discussion

NEXGB is inspired by the latest advances in network medicine [21,22]. To better learn the potential information between nodes in the PPI network, we use struc2vec to explore the synergy between anticancer drugs, drugs, and cell lines. The input features of XGBoost are the vector combinations of drugs and cell lines, and the output is the predicted probability of drug combinations. In contrast to other models, NEXGB does not use detailed chemical and biological descriptions (genomic profiles). It is only necessary to consider the combinations of drug–target and cancer cell line–target and their topological relationships in the PPI network. We apply NEXGB to the Oncology-Scree and DrugCombDB datasets and demonstrate the superiority of NEXGB. NEXGB has discovered potential relationships between drug combinations and cell lines. Based on the performance of the model on a specific cell line, we speculate that the ES2 cell line differs from other cell lines and has unique properties among ovarian-cancer-related cell lines. Overall, NEXGB can attempt to identify novel combination therapies for other intractable diseases based on biological knowledge in the PPI network. This adaptability to other diseases still requires further exploration. The model provides new ideas for medical fields such as cancer therapy.

With the improvement of more biological network data, we will be able to acquire more network information to train NEXGB. However, the prescription of clinical combinations requires knowledge of pharmacokinetic interactions between drugs. Personalized treatment needs to take into account the individual conditions of different patients, such as tolerance to the drug and unique genomic information. These factors are mostly ignored by NEXGB and other existing methods. The increase in available information brings corresponding problems. Different measurement standards in the same drug combination leads to different results. How to balance these biological experimental data is also a critical problem. Nonetheless, we believe that further studies in pharmacology, pharmacokinetics, toxicology, and genetic heterogeneity, accompanied by new computational methods, can rapidly overcome these limitations.

## 4. Datasets and Methods

### 4.1. Datasets

Drug–Drug–Cell Line synergy. We evaluate NEXGB on two anti-cancer drug combination datasets: (a) Oncology-Screen [28], an oncology screening dataset (accessed on 25 August 2021) including 21 drugs and 29 tumor cell lines, with a total of 4176 combinations, using Loewe values as the synergy score; (b) DrugCombDB [29], a much larger dataset, which is currently the largest database (accessed on 12 September 2021) for the number of drug combinations, containing 764 unique drugs and 69,436 drug combinations in 76 unique cell lines, using ZIP values as the synergy score. In the two datasets, we divide the positive and negative samples according to the synergy value. A synergy value greater than zero indicates a positive sample, and a synergy value less than zero indicates a negative sample. The number of positive and negative samples in Oncology-Scree is 2257 and 1919, respectively, while there are 31,623 positive samples and 37,813 negative samples in DrugCombDB.

Protein–Protein Interaction (PPI) Network. The PPI network (accessed on 17 September 2021) contains 15,970 nodes (unique proteins) and 217,160 edges (interactions). The network is composed of 15 commonly used databases and experimental evidence [22,25]. Proteins are represented using gene numbers, with the coding mapped by GeneCards.

Drug–Protein Associations. A total of 15,051 drug–protein associations are established based on FDA-approved or clinically investigated drugs [22]. The dataset contains 4428 drugs and 2256 unique human proteins (accessed on 17 September 2021).

Cell–Protein Associations. The cell–protein association dataset was obtained from the Cancer Cell Line Encyclopedia (CCLE) [37]. This dataset exists for 749,551 associations, 1035 cancer cell lines, and 18,022 proteins (accessed on 20 September 2021).

Related information is available at https://github.com/lysmfj/NEXGB (accessed on 30 September 2021).

### 4.2. Framework of NEXGB

**Figure 5:**
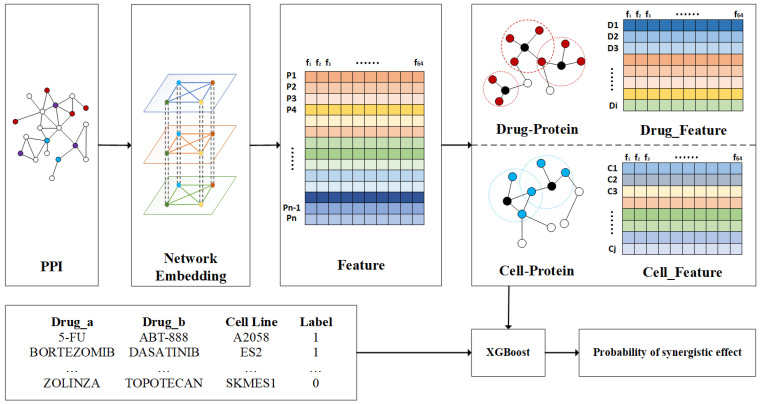
The framework of the NEXGB. The PPI network serves as the input of the model, where red represents the drug target protein, blue is the cell line target protein, and purple is the drug–cell line interaction protein. The struc2vec component perform feature extraction for the protein nodes in the input PPI network. We further obtain the features of drugs and cell lines through the drug–protein and cell–protein relationships. Labels 1 and 0 in the drug–drug–cell line combination matrix indicate synergistic and antagonistic effects, respectively. The drug characteristics and cell line characteristics are then concatenated and input into XGBoost. The output of XGBoost is the combined synergistic probability.

The related process of NEXGB is illustrated in Figure 5. NEXGB takes a drug–drug–cell line combination as input and outputs a predicted probability of the synergy value of the combination. The input to the PPI network of the struc2vec component is the coding gene sequence number, and the output is the 64-dimensional latent feature $f_{64}$ of n proteins. The target protein data for drug and cell line effects are similar to the PPI network data. We take the mean value of the characteristics of the proteins directly affected by the drug (the same is true for the cell line characteristics), and the characteristics of the drug $D_{i}$:

(1) $$D_{i}=\frac{P_{1}+P_{2}+\cdots \cdots +P_{n}}{n}$$

We further formulate the synergistic drug combination prediction problem as a binary classification problem. According to the definition of the synergy score, a synergy value greater than 0 indicates a synergistic effect between two drugs [38]. We regard a synergy value greater than zero as a positive sample (label: 1) in the drug combination data, and a value less than zero as a negative sample (label: 0). We concatenate the obtain drug features and cell line features according to the drug–drug–cell line combination data, input the combined features and labels into XGBoost for training, and output the prediction results.

### 4.3. Network Embedding

In the PPI network, a node represents the gene encoding of a protein, and the existence of an edge between two nodes indicates that the two proteins interact. PPI networks are an undirected graph. We utilize the struc2vec [26] model in the network embedding approach to learn the structural features of the PPI network, using a vector to represent the genes in the network. The struc2vec model encodes structural similarities by building multi-layer graphs and generates structural contexts for nodes. Gene pairs that are far apart but structurally similar are tightly embedded in the PPI network. The main steps of the struc2vec component are as follows:

#### 4.3.1. Structural Similarity

The calculation of the structural similarity $f\left(u,v\right)$ between each pair of nodes u and v can be denoted as:

(2) $$f_{k}\left(u,v\right)=f_{k-1}\left(u,v\right)+g\left(s\left(R_{k}\left(u\right)\right),s\left(R_{k}\left(v\right)\right)\right)$$

where $f_{-1}=0$, $R_{k}(u)$ is the set of nodes with distance $k\left(k\geq 0\right)$ from node u, $s\left(R_{k}\left(u\right)\right)$ is the ordered degree sequence of node set $R_{k}(u)$, and $g\left(s\left(R_{k}\left(u\right)\right),s\left(R_{k}\left(v\right)\right)\right)>0$ is a measure of the distance between the ordered sequences $s\left(R_{k}\left(u\right)\right)$ and $s\left(R_{k}\left(v\right)\right)$.

In general, $s\left(R_{k}\left(u\right)\right)$ and $s\left(R_{k}\left(v\right)\right)$ are always of different sizes, and the node degrees are integers. In order to compare such two-degree sequences of different size, we use dynamic time warping (DTW) [39]:

(3) $$g\left(s\left(R_{k}\left(u\right)\right),s\left(R_{k}\left(v\right)\right)\right)=\frac{\max\left(s\left(R_{k}\left(u\right)\right),s\left(R_{k}\left(v\right)\right)\right)}{\min\left(s\left(R_{k}\left(u\right)\right),s\left(R_{k}\left(v\right)\right)\right)}-1$$

#### 4.3.2. Construction of Multilayer Weighted Graph

Each layer $k=0,\ldots ,k^{*}$ consists of a weighted undirected graph, and the edge weight between nodes u and v in the same layer is defined as:

(4) $$w_{k}\left(u,v\right)=e^{-f_{k}\left(u,v\right)},k=0,1,\ldots ,k^{*}$$

where $k^{*}$ is the diameter of the original network. In the k layer, each node u is connected to the corresponding node u in the k+1 layer and k−1 layer. The weights of the edge between different layers are defined as:

(5) $$\begin{array}{l} w\left(u_{k},u_{k+1}\right)=\log\left(\Gamma_{k}\left(u\right)+e\right)\\ w\left(u_{k},u_{k-1}\right)=1 \end{array}$$

where $\Gamma_{k}\left(u\right)$ is number of edges incident to u that have weight larger than the average edge weight of the complete graph in layer k.

#### 4.3.3. Generation of Node Sequence

A biased random walk is applied to generate a sequence of nodes in a multi-layer graph. When performing a random walk, staying at the current layer with the probability q, the probability from node u to node v in the k-th layer is:

(6) $$p_{k}\left(u,v\right)=\frac{e^{-f_{k}\left(u,v\right)}}{Z_{k}\left(u\right)}$$

where $z_{k}\left(u\right)=\sum_{v¹u}e^{-f_{k}\left(u,v\right)}$ is the normalization factor for vertex u in layer k. The random walk switches to other layers with probability 1−q, and the probability of selecting layer k+1 and layer k−1 is as follows:

(7) $$\begin{array}{l} p_{k}\left(u_{k},u_{k+1}\right)=\frac{w\left(u_{k},u_{k+1}\right)}{w\left(u_{k},u_{k+1}\right)+w\left(u_{k},u_{k-1}\right)}\\ p_{k}\left(u_{k},u_{k-1}\right)=\frac{w\left(u_{k},u_{k-1}\right)}{w\left(u_{k},u_{k+1}\right)+w\left(u_{k},u_{k-1}\right)} \end{array}$$

when generating the node sequence, use the Skip-Gram [40] model to train the node sequence to generate the node vector.

### 4.4. Supervised Classification Model

XGBoost is an improvement of the gradient boosting algorithm, and the internal decision tree uses a regression tree [27]. We apply extreme gradient boosting (XGBoost) in supervised learning to classify based on constructed drug–cancer cell line features to predict their synergistic relationships.

## 5. Conclusions

In this study, we propose a novel network embedding model NEXGB for predicting the relationships between drug combinations and cancer cell lines in cancer. The results show that NEXGB can effectively identify anticancer drug combinations through the topological features of the PPI network, the biological mechanisms of drug-related proteins, and proteins associated with cancer cell lines. In addition, the results on specific cell lines suggest that ES2 cell lines may have unique biological properties among ovarian cancer cell lines.

## Figures and Tables

**Table 1:** The key parameters of the network embedding method.

Network Embedding	
the number of the random walk starts for each gene	20
the length of each random walk sequence	80
the number of layers	4
the context size for optimization	5
the dimension of the node vector	64

**Table 2:** The key parameters of the extreme gradient boosting method.

XGBoost	
booster	gbtree
max_depth	7
min_child_weight	1
gamma	0.1
subsample	0.9
colasmple_bytree	0.9
learning_rate	0.1

**Table 3:** The performance of NEXGB compared with baselines.

Model	Oncology-Screen					DrugCombDB				
	ACC	Recall	AUC-ROC	AUC-PR	F1	ACC	Recall	AUC-ROC	AUC-PR	F1
NP	0.455	0.064	0.489	0.535	0.112	0.674	0.175	0.475	0.446	0.243
GraRep	0.663	0.767	0.723	0.702	0.702	0.667	0.581	0.728	0.689	0.613
DeepWlak	0.683	0.741	0.743	0.765	0.723	0.674	0.596	0.736	0.698	0.628
DeepSynergy	0.689	0.718	0.766	0.769	0.705	0.685	0.626	0.746	0.729	0.643
GraphSynergy	0.762	0.782	0.847	0.855	0.774	0.753	**0.714**	**0.834**	**0.815**	0.727
NEXGB	**0.782**	**0.827**	**0.858**	**0.873**	**0.804**	**0.762**	0.704	0.833	0.811	**0.729**

The data with the highest score are listed in bold for readability.

**Table 4:** The number of target proteins associated with each cancer cell.

**Cell Line**	**Protein Quantity**	NCIH460	305
KPL1	369	NCIH520	602
MDAMB436	390	SKMES1	208
T47D	463	A2780	480
ZR751	679	CAOV3	282
DLD1	75	ES2	56
HCT116	117	OV90	505
HT29	379	SKOV3	328
LOVO	132	VCAP	830
RKO	147	A2058	339
SW620	381	A375	156
SW837	533	HT144	147
NCIH1650	122	RPMI7951	142
NCIH2122	346	SKMEL30	107
NCIH23	136	UACC62	125

## Data Availability

Not applicable.
